# Prevalence, Clinical Characteristics, and Therapeutic Underachievement in Familial Hypercholesterolemia: Results from the GRegistry-FH Population-Based Study in Greece

**DOI:** 10.3390/jcm15135127

**Published:** 2026-07-01

**Authors:** Genovefa Kolovou, Stamatis Makrygiannis, Niki Pavlatou, Christina Marvaki, Olga Kadda, Aikaterini Marvaki, Petros Kalogeropoulos, Vana Kolovou, Anastasios Tzenalis, Zeimpek Emre, Edison Jahaj, Zoi Kasiara, Ilias Giannakoulis, Ioannis Tsolakoglou, Nikolaos Tsaloukidis, Rafailia Koulaxidou, Katherine Anagnostopoulou, Vasiliki Giannakopoulou, Georgios Goumas, Sotiria Limberi, Despina Perrea, Olga Ampartzidou, Dimitrios Kosmidis, Maria Stravogianni, Athanasia Striki, Michael I. Kourakos, Ioannis Hoursalas, Charalambos Vlachopoulos, Loukianos Rallidis, Niki Katsiki, Andreas Melidonis, Stefanos Foussas, Haralampos Milionis, Evaggelos Liberopoulos, Helen Bilianou

**Affiliations:** 1 Preventive Cardiology, Lipoprotein Apheresis and Lipid Disorders Clinic, Cardiometabolic Center, Metropolitan Hospital, 9 Ethn. Makariou& 1 El Venizelou Str, 185 47 Athens, Greece; petros.kalo@gmail.com (P.K.);; 2 First Department of Cardiology, “Hygeia” Diagnostic and Therapeutic Centre of Athens, 151 23 Athens, Greece; ssmakrygiannis@gmail.com; 3 Pathological and Surgical Nursing, University of West Attica, 115 27 Athens, Greece; 4 Department of Nursing, University of West Attica, 190 03 Athens, Greece; 5 Department of Electrophysiology and Pacing, Onassis Cardiac Surgery Centre, 174 55 Athens, Greece; 6 Katerini Hospital, 601 33 Katerini, Greece; 7 Pathological Nursing/Intensive Care Unit, University of Patras, 570 13 Patra, Greece; 8 Radiology Department, Hospital of Xanthi, 671 00 Xanthi, Greece; 9 Dermatology Department, Evangelismos General Hospital, 106 76 Athens, Greece; 10 Sports and Health, School of Medicine & School of Physical Education and Sport Science, Aristotle University of Thessaloniki, 731 00 Thessaloniki, Greece; 11 Argolidas General Hospital, 212 31 Argos, Greece; 12 General Hospital of Thessaloniki ‘Agios Pavlos’, 551 34 Thessaloniki, Greece; 13 Department of Nursing, University General Hospital ‘ATTIKO’, 104 42 Athens, Greece; 14 Biomedical Methods and Technology in Diagnosis, 104 38 Athens, Greece; 15 Molecular Genetics and Genomics Department, Medical Neoscreen Ltd., 152 35 Athens, Greece; kat_anag@yahoo.com; 16 Department of Cardiology, Tzanio Hospital, 185 36 Piraeus, Greece; 17 Cardiology Clinic, Athens Euroclinic, 115 21 Athens, Greece; ggoumasgr@yahoo.gr; 18 Department of Cardiology, Sotiria Hospital, 115 27 Athens, Greece; 19 Laboratory of Experimental Surgery and Surgical Research ‘N.S. Christeas’, Medical School, National and Kapodistrian University of Athens, 115 27 Athens, Greece; 20 Department of Internal Medicine, Metropolitan Hospital, 185 47 Athens, Greece; 21 Department of Nursing, Democritus University of Thrace, 642 00 Alexandroupolis, Greece; 22 Anesthesiology Department, Katerini’s General Hospital, 601 32 Katerini, Greece; 23 General Hospital of Athens “G. Gennimatas”, 115 27 Athens, Greece; 24 Department of Nursing, School of Health Sciences, University of Ioannina, 455 00 Ioannina, Greece; 25 School of Medicine, European University Cyprus, 220 25 Nicosia, Cyprus; 26 First Department of Cardiology, Medical School, National and Kapodistrian University of Athens, Hippokration Hospital, 115 27 Athens, Greece; 27 Department of Cardiology, Medical School, National and Kapodistrian University of Athens, Attikon Hospital, 124 61 Athens, Greece; lrallidis@gmail.com; 28 Department of Nutritional Sciences and Dietetics, International Hellenic University, 574 00 Thessaloniki, Greece; 29 Metropolitan Hospital, Cardiometabolic Center, 185 47 Athens, Greece; 30 Cardiology Department, Metropolitan Hospital General, 155 62 Athens, Greece; 31 Department of Internal Medicine, School of Medicine, University of Ioannina, 451 10 Ioannina, Greece; hmilioni@uoi.gr; 32 First Department of Propaedeutic Internal Medicine, Medical School, Laiko General Hospital, National and Kapodistrian University of Athens, 115 27 Athens, Greece; vaglimp@yahoo.com; 33 Hellenic College of Atherosclerosis Treatment, Cardiology Clinic, “Tzaneio” General Hospital, 185 36 Piraeus, Greece

**Keywords:** familial hypercholesterolemia, LDL cholesterol, Greece, cardiovascular risk, lipid-lowering therapy

## Abstract

**Background and Aim**: Familial hypercholesterolemia (FH) is a genetic disorder leading to severely elevated LDL-C and premature atherosclerotic cardiovascular disease (ASCVD). This study, the GRegistry-FH, provides the first population-based estimation of heterozygous FH (HeFH) prevalence and the clinical profile of affected individuals in Greece. **Methods**: A cross-sectional, questionnaire-based study was conducted on a representative sample of 7704 adults across 22 Greek regions. HeFH was assessed using the Simon Broome and Dutch Lipid Clinic Network (DLCN) criteria. Clinical characteristics and lipid-lowering therapy (LLT) attainment were compared between individuals with the HeFH phenotype and the general population. **Results**: The prevalence of HeFH was estimated at 1 in 188 individuals (Simon Broome) and 1 in 183 (DLCN). Individuals with the HeFH phenotype were significantly older and exhibited a much higher prevalence of hypertension (48.8% vs. 23.9%). Notably, individuals with the HeFH phenotype experienced myocardial infarction an average of 14 years earlier than non-FH individuals. Although 80.5% of individuals with the HeFH phenotype were on LLT, only 18.2% achieved LDL-C goals. None of the individuals with the HeFH phenotype with established ASCVD reached the target LDL-C of <1.4 mmol/L (55 mg/dL). **Conclusions**: HeFH is highly prevalent in Greece but remains largely underdiagnosed in younger ages and suboptimally treated. Despite high treatment rates, the vast majority of individuals with the HeFH phenotype fail to reach protective LDL-C targets. These findings emphasize the need for earlier identification and more aggressive combination lipid-lowering strategies.

## 1. Introduction

Familial hypercholesterolemia (FH) is a genetic condition with high penetrance that leads to severely elevated plasma low-density lipoprotein cholesterol (LDL-C). This metabolic derangement is a primary driver of premature atherosclerotic cardiovascular disease (ASCVD), particularly manifesting as coronary artery disease (CAD) and carotid artery stenosis [1,2,3,4]. While the prevalence of heterozygous FH (HeFH) was historically thought to be 1 in 500, modern genetic research in North American and European cohorts suggests the actual frequency may exceed 1 in 250 individuals [5].

These prevalence estimates can vary significantly depending on the diagnostic criteria employed and the specific population being studied. Currently, HeFH is estimated to affect between 1 in 200 and 1 in 250 people, making it the most prevalent monogenic disorder identified to date [2,3,4,5,6,7,8,9,10]. In contrast, homozygous FH (HoFH) is much rarer, appearing in approximately 1 per 160,000–300,000 individuals [2,11,12]. The genetic basis for HoFH includes true homozygosity (the same mutation on both alleles), bi-allelic semi-dominant hypercholesterolemia (different mutations on each allele of the same gene), or digenic inheritance (mutations in two different genes, such as *LDLR* and *APOB*, that both impair LDL receptor function) [2].

The clinical presentations of HeFH and HoFH frequently overlap. This phenotypic variability is driven by several factors, including the heterogeneity of monogenic causes, the influence of polygenic variants, gene–environment interactions, and epigenetic modifications. Furthermore, an individual’s plasma LDL-C level is the result of both high-impact monogenic mutations and the cumulative effect of various small-effect genetic variants [13,14].

The prognosis for untreated HeFH is concerning: roughly 50% of men and 30% of women will experience a myocardial infarction (MI) before the ages of 50 and 60, respectively [15,16,17,18]. With an estimated 35 million people affected worldwide, FH represents a major public health crisis. Current data suggest that only about 10% of these individuals have been formally diagnosed, and over 80% of those receiving treatment fail to achieve recommended LDL-C targets. Conversely, intensive LDL-C reduction has been proven to significantly lower ASCVD events in this population [3,19].

Therefore, improving the identification of FH phenotypes and increasing clinical awareness is essential. If left unmanaged, the disease carries an ASCVD burden approximately 20 times higher than that of the general population [20]. National registries are indispensable tools in this effort; they provide the data necessary to guide prevention strategies, optimize treatment protocols [16], and educate healthcare providers. As Greece currently lacks population-based prevalence data for FH, this study aims to determine the prevalence and clinical characteristics of HeFH within the Greek general population.

## 2. Methods

### 2.1. Study Design and Population

The GRegistry-FH is a cross-sectional, questionnaire-based study focused on the general population, organized by the Hellenic College of Treatment of Atherosclerosis (HCTA) [ClinicalTrials.gov, https://clinicaltrials.gov, NCT03140605, registered on 5 May 2017] [21]. The study protocol received approval from the Medical School of the National and Kapodistrian University of Athens (nr. 1516016332/09.02.2016). While the full rationale and methodology have been published previously, the study briefly involved partitioning Greece into 22 geographical sectors (including 8 in the Athens metropolitan area) based on the 2011 Hellenic General Population Census (ELSTAT) [21].

Trained interviewers conducted face-to-face assessments within each allocated region. Notably, the interviewers were educated by lipid specialists, attended outpatient lipid clinics, and watched slides with FH cases with xanthomas. Participants were required to be over 18 years of age; those unable or unwilling to provide informed consent were excluded. A total of 349 individuals (339 men) declined to participate. All enrolled subjects provided written informed consent. The recruitment started in January 2017 and was scheduled to be concluded by the end of 2020. However, the COVID-19 pandemic was a major setback due to the nature of the study, which prolonged our sampling period until September 2023.

### 2.2. Data Collection and Variables

Upon completion, the principal investigator reviewed a predetermined number of questionnaires for accuracy. Data were managed via two separate files—one for contact details and another for clinical information—linked by a unique identification number to ensure anonymity during analysis in SPSS 26 (IBM Corp., Armonk, NY, USA). The survey utilized a comprehensive questionnaire covering:

- Demographics: age, sex, height, weight, and body mass index (BMI).
- Medical History: personal and family history of premature ASCVD and cardiovascular risk factors.
- Clinical Data: blood pressure readings and laboratory values for glucose and lipid parameters.
- Lifestyle: smoking status (current, ex-smoker, or non-smoker), dietary habits, and physical activity.

Comprehensive data regarding the entire cohort’s risk factors and baseline characteristics have been detailed by Kolovou et al. [22].

### 2.3. Diagnostic Criteria for HeFH

The prevalence and clinical profile of HeFH were primarily assessed using the Simon Broome Register criteria. For supplementary validation, an additional assessment was conducted using the Dutch Lipid Clinic Network (DLCN) criteria [23].

### 2.4. Simon Broome Register Criteria

Participants were classified as having “Definite” or “Possible” FH based on LDL-C levels, physical markers, genetic data (if available), and family history [24]:

- Definite FH: LDL-C > 4.9 mmol/L (190 mg/dL) PLUS the presence of tendon xanthomas (in the patient or a relative) OR evidence of a causative genetic mutation (*LDLR*, *APOB*, or *PCSK9*).
- Possible FH: LDL-C > 4.9 mmol/L (190 mg/dL) PLUS a family history of premature MI or elevated total cholesterol (>7.5 mmol/L).

### 2.5. Dutch Lipid Clinic Network (DLCN) Criteria

This scoring system categorizes diagnoses based on a cumulative point scale [23]:

- Definite FH: >8 points;
- Probable FH: 6–8 points;
- Possible FH: 3–5 points;
- Unlikely FH: 0–2 points.

Points are awarded across five groups: family history (Group 1), clinical history of premature CAD or vascular disease (Group 2), physical examination findings like corneal arcus or xanthomas (Group 3), biochemical LDL-C levels (Group 4), and molecular genetic findings (Group 5).

### 2.6. Statistical Analysis

All participants required documented LDL-C levels. For those on lipid-lowering therapy (LLT), both pre-treatment and post-treatment values were recorded. Definitions for hypertension, diabetes, and dyslipidemia followed the 2019 ESC/EAS Guidelines [25].

Sample size was determined by an a priori power analysis (power = 0.80; CI = 0.95), assuming a margin of error of 0.001 based on previous prevalence estimates. Continuous variables are reported as mean (SD) and categorical variables as percentages. Comparisons were performed using independent-sample *t*-tests or Mann–Whitney U tests for continuous data and chi-square or Fisher’s exact tests for categorical data. Analysis was conducted using IBM SPSS version 26.0, with statistical significance set at *p* < 0.05.

## 3. Results

### 3.1. Prevalence of HeFH in the Greek Population

The final analysis of the GRegistry-FH included a total of 7704 individuals. Within this cohort, 3.0% (*n* = 234) exhibited total cholesterol levels exceeding 7.5 mmol/L (290 mg/dL), and 2.8% (*n* = 214) presented with LDL-C levels above 4.9 mmol/L (190 mg/dL), with typical overlapping observed between these two groups.

Based on the pre-specified Simon Broome Register criteria, 41 individuals were identified as having “definite” (5 out of 41) or “possible” FH (36 out of 41). Simultaneously, the DLCN criteria identified 42 individuals meeting the “definite” or “probable” thresholds (12 and 30 out of 42, respectively). Consequently, the estimated prevalence of HeFH in Greece is 1 in 188 individuals (0.5%, 95% CI 0.4% to 0.7%) based on the primary criteria. It is noteworthy that the two diagnostic sets concurred on only 30 specific cases; if a diagnosis is considered valid by meeting either set of criteria, the total count rises to 53 patients, suggesting a potential prevalence as high as 1 in 145.

Of the identified cases, two individuals displayed extreme LDL-C elevations (>10.2 mmol/L or 400 mg/dL), though neither fulfilled the additional clinical criteria for a homozygous FH (HoFH) diagnosis. Regarding geographical distribution, nearly half of the cases (48.8%; *n* = 20) were concentrated in Attika and Central Macedonia, mirroring the population density of Greece’s two largest urban centers.

### 3.2. Clinical and Risk Factor Profile

The clinical characteristics of individuals with the HeFH phenotype were compared against the general study population. On average, those identified with HeFH were significantly older than non-HeFH participants (55.7 ± 12.6 vs. 49.6 ± 17.1 years; *p* = 0.004). Furthermore, individuals with the HeFH phenotype demonstrated a markedly higher prevalence of ASCVD and myocardial infarction (MI).

A sub-analysis of patients with established ASCVD revealed that those with HeFH developed the disease much earlier:

- Mean Age of ASCVD: 55 ± 14 years for HeFH vs. 67 ± 12 years for the general population (*p* < 0.001).
- Premature Manifestation: 82.4% (14 out of 17) of individuals with the HeFH phenotype with ASCVD experienced premature onset.
- Myocardial Infarction: The mean age for MI in the HeFH group was 52 ± 12 years compared to 66 ± 13 years in others (*p* < 0.001), with 90% of these events classified as premature.

HeFH individuals were also significantly more likely to report a family history of dyslipidemia and ASCVD. Additionally, hypertension was significantly more frequent in the HeFH group (48.8% vs. 23.9%, *p* < 0.001), while current smoking was non-significantly higher (41.5% vs. 28.9%, *p* = 0.07), and the prevalence of diabetes mellitus did not differ significantly between groups (*p* = 0.18).

Table 1 summarizes the baseline characteristics of both the general population and individuals with the HeFH phenotype (Table 1).

**Figure 1:**
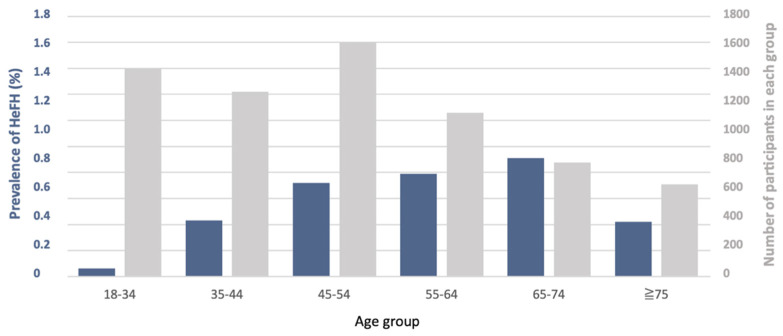
Prevalence of heterozygous familial hypercholesterolemia (HeFH) (blue bars) and number of participants (grey bars) across age groups.

Figure 1 shows the prevalence of HeFH and the number of participants across age subgroups.

### 3.3. Lipid-Lowering Therapy (LLT) and Target Attainment

As expected, individuals with the HeFH phenotype maintained significantly higher mean LDL-C values than the rest of the cohort, even after treatment. While 80.5% of HeFH individuals were receiving LLT—a rate significantly higher than the 19.8% observed in the general population (*p* < 0.001)—therapeutic goal attainment remained poor.

Plasma LDL-C concentrations in individuals with the HeFH phenotype decreased by an average of 42.5 ± 18.7%, reaching a mean post-treatment value of 3.3 ± 1.35 mmol/L (130 ± 53 mg/dL). While this reduction was statistically more significant than the 29.8 ± 20.2% decrease seen in the general population (*p* = 0.048), only 18.2% of treated HeFH individuals reached their specific therapeutic goal. Furthermore, the percentage of individuals with the HeFH phenotype achieving an LDL-C below 100 mg/dL tended to be lower, though not significantly, than the non-HeFH individuals (24.2% vs. 38.7%), as did the percentage reaching below 70 mg/dL (6.1% vs. 9.8%). Notably, within the subgroup of individuals with the HeFH phenotype and established ASCVD, none (0%) achieved the stringent goal of LDL-C below 1.4 mmol/L (55 mg/dL).

Table 2 summarizes lipid, glucose, and blood pressure measurements in the general population and in individuals with the HeFH phenotype.

## 4. Discussion

The cornerstone finding of the GRegistry-FH study is a calculated HeFH prevalence of 1 in 188 within the Greek population using Simon Broome criteria. This is the first study of its kind to bypass specialty lipid clinics and sample the general Greek population directly, providing a highly accurate snapshot of the national disease burden. This 1:188 figure already suggests a higher prevalence than historical estimates, highlighting the need for further research in real-world settings. When using a composite diagnostic approach—considering any patient who met either the Simon Broome or DLCN thresholds—the prevalence rose to 1 in 145.

A critical nuance of our data is the “age-dependent diagnostic gap.” We observed a significantly lower prevalence in participants under age 40 (1 in 2205), despite the genetic nature of the disease, which dictates a constant prevalence across all age brackets. This discrepancy highlights a fundamental flaw in current clinical scoring systems: they are heavily weighted toward cumulative “damage” (e.g., higher LDL-C over time, presence of physical signs, or established ASCVD). Consequently, younger patients—who would benefit most from primordial prevention—are systematically under-detected. This is consistent with the Simon Broome registry follow-up data, showing that only 25% of affected individuals are diagnosed by middle age [24].

The Greek prevalence of 1:188 aligns with the emerging global consensus that FH is far more common than the traditional 1:500 estimate. However, geographic and ethnic variability remains stark due to founder effects. While our findings mirror other Mediterranean populations like the Catalan cohort (1:192) [26], they stand in contrast to high-prevalence “islands” such as the French Canadians (1:270), South African Afrikaners (1:72), and Christian Lebanese (1:85) [27,28,29,30].

Our data also contribute to the understanding of the complex genetic architecture of FH. While we identified two individuals with extreme LDL-C elevations (>10.2 mmol/L), neither met the full clinical criteria for homozygous FH (HoFH). This highlights the phenotypic overlap between severe HeFH and HoFH, often driven by bi-allelic semi-dominant or digenic mutations [2]. Understanding that at least 3000 mutations exist within the *LDLR*, *APOB*, and *PCSK9* genes underscores why clinical presentation remains the pragmatic “gold standard” for diagnosis, even in the absence of genetic testing [2].

Perhaps the most provocative finding is the high rate of lipid-lowering therapy (LLT) usage (80.5%) coupled with dismal target attainment. This suggests that while Greek physicians are active in prescribing statins, they may not be identifying these patients as “FH cases,” leading to a lack of therapeutic intensity.

Despite an average LDL-C reduction of 42.5% in the HeFH group, the mean post-treatment value remained significantly higher than that of the general population (3.3 mmol/L vs. 2.76 mmol/L). Furthermore, the fact that none of the ASCVD patients reached the <1.4 mmol/L (55 mg/dL) target might indicate a “stagnation” in treatment titration. This “therapeutic inertia” is a global phenomenon; even in the Netherlands, only 12.2% of genetically defined FH patients reach targets [31]. Our results emphasize that for HeFH, statin monotherapy is often insufficient. The rigorous application of high-intensity statins combined with ezetimibe and PCSK9 inhibitors—as validated by the IMPROVE-IT [32] and ODYSSEY [33] trials—must become the standard of care to bridge this gap.

FH does not exist in a vacuum; its cardiovascular risk is compounded by co-morbidities. In our study, HeFH individuals were not only burdened by genetic cholesterol but also by a significantly higher prevalence of hypertension (48.8%), a finding also reported in the HELLAS-FH registry [34]. The percentage of active smokers was also high (41.5%), although it was not found to be significantly higher compared with the general population. This observation is particularly concerning, as tobacco use and LDL-C elevations act synergistically to accelerate endothelial dysfunction. The observed 7.9-fold higher prevalence of MI, which occurs an average of 14 years earlier than in the general population, may be attributed to the observed risk factor burden. While some studies suggest FH might offer a “protective” effect against diabetes [35], our data showed a high, non-significant prevalence of DM (17.1%), likely due to the older mean age of our cohort that came from the general population. It is of interest that, in contradiction to cohorts that come from specialty centers and include both the index and the typically younger non-index cases, a primary care-based study with a mean age comparable to that in our data reported an even significantly higher prevalence of diabetes in the HeFH phenotype group [26]. These findings reinforce the need for a holistic cardiovascular management strategy in FH patients that goes beyond just LDL-C lowering towards aggressive risk factor control.

The prediction by Goldstein et al. [36] that 1 in 160 individuals carry a major genetic lipid disorder is remarkably prescient when viewed against our data. National registries like GRegistry-FH are vital for moving the needle from reactive treatment to proactive screening. Whether through child–parent screening (effective between ages 1–9) or family cascade testing, the identification of these 35 million individuals worldwide remains one of the most cost-effective ways to reduce the global burden of premature ASCVD [37].

### Strengths and Limitations

The primary strength of the GRegistry-FH study lies in its design as the first large-scale, population-based effort to quantify the prevalence of FH in Greece. Unlike many previous registries that recruit exclusively from specialized lipid clinics—which naturally creates a selection bias toward severe cases—this study sampled the general adult population. To ensure a representative cohort, the recruitment strategy mirrored the geographical and demographic distribution of the official national census, deliberately avoiding healthcare facilities to capture “real-world” data.

The meticulous training of interviewers and the high level of participant engagement resulted in a comprehensive dataset. This robust sample size allowed for a detailed characterization of the HeFH clinical phenotype and permitted statistically significant comparisons against the non-FH general population, providing a clear picture of the relative cardiovascular burden in Greece.

Despite these strengths, several limitations must be acknowledged. First, the absence of molecular genetic testing means that pathogenic variants in the *LDLR*, *APOB*, or *PCSK9* genes were not confirmed. While clinical criteria are the standard for bedside diagnosis, it is estimated that only 40–50% of patients meeting clinical FH criteria possess an identifiable monogenic mutation. Consequently, some cases might represent polygenic hypercholesterolemia rather than monogenic FH.

Second, the study did not account for the contribution of Lipoprotein(a) [Lp(a)] to the measured LDL-C levels. Elevated Lp(a) can lead to an overestimation of LDL-C, and research suggests that up to 3% of FH patients might be reclassified to a lower diagnostic category if Lp(a) corrected LDL-C were utilized [38]. It should be emphasized that the absence of genetic testing and Lp(a)-corrected LDL-C could have influenced case classification and prevalence estimation. However, measuring Lp(a) (and, of course, genetic testing) requires specialized laboratory assays that were not accessible for a general population screening of this scale.

Third, the cross-sectional nature of the study limits our ability to establish temporal or causal relationships between variables. Fourth, the data collection period was extended due to the COVID-19 pandemic, which may have introduced temporal changes in the recorded variables. Additionally, the reliance on self-reported data for medical history, smoking status, and family history introduces potential recall bias. While we mitigated this by reviewing biochemical reports and prescriptions whenever available, some inaccuracies may persist. Furthermore, a higher participation rate among women was observed, which requires cautious interpretation of gender-specific data, although FH itself is not gender-linked. We should take into consideration that some of the differences regarding hypertension, DM, and ASCVD events may be due to age or other confounding factors. Finally, data on specific lipid-lowering drug treatments were not analyzed in the current study. However, a separate comprehensive analysis with more details on lipid-lowering agents, titration, and escalation for both HeFH and non-HeFH individuals will be conducted in the future.

## 5. Conclusions

The GRegistry-FH study establishes that the prevalence of heterozygous FH in Greece is approximately 1 in 188, a figure higher than historical projections and consistent with modern European data. Despite the genetic nature of the disorder, a significant “diagnostic gap” exists in younger populations, leading to late-stage identification only after the appearance of clinical markers or cardiovascular events.

The most critical clinical insight is the existence of therapeutic inertia in terms of achieving LDL-C goals: despite high rates of treatment (80.5%), a vast majority of individuals with the HeFH phenotype fail to reach recommended LDL-C targets, particularly those with established ASCVD. This underscores the need for more aggressive, multi-drug lipid-lowering strategies and intensified public health initiatives. By increasing clinical awareness and implementing systematic screening, we can shift from reactive management to early intervention, ultimately reducing the 20-fold higher cardiovascular burden faced by this population.

## Figures and Tables

**Table 1:** Baseline characteristics of the heterozygous familial hypercholesterolemia (HeFH) phenotype population and the general population.

	General Population	HeFH Phenotype	*p*
*n*	7663	41	
Men, *n* (%)	3238 (42.3)	23 (56.1)	0.074 (ns)
Women, *n* (%)	4425 (57.7)	18 (43.9)	
Age, years mean (SD)	49.6 (17.1)	55.7 (12.6)	0.004
ΒΜΙ, kg/m^2^ mean (SD)	26.5 (6.7)	26.4 (3.8)	0.9 (ns)
Current smoking, *n* (%)	2212 (28.9)	17 (41.5)	0.07 (ns)
Hypertension, *n* (%)	1830 (23.9)	20 (48.8)	<0.001
Diabetes mellitus, *n* (%)	810 (10.6)	7 (17.1)	0.18 (ns)
Pre-diabetes mellitus *, *n* (%)	1419 (25.4)	5 (16.1)	0.23 (ns)
Personal history of MI, *n* (%)	238 (3.1)	10 (24.4)	<0.001
Personal history of CHD, *n* (%)	445 (5.8)	17 (41.5)	<0.001
Personal history of stroke, *n* (%)	357 (4.7)	8 (19.5)	<0.001
Relatives with hypercholesterolemia, mean (SD)	1 (1)	3 (1)	<0.001
Family history of CHD, *n* (%)	2452 (32.0)	27 (65.9)	<0.001
Family history of stroke, *n* (%)	2158 (28.2)	16 (39.0)	0.12 (ns)

BMI, body mass index; CHD, coronary heart disease; MI, myocardial infarction; SD, standard deviation; ns, non-significant. * Percentages based upon 5588 participants with relevant data.

**Table 2:** Measurements of the heterozygous familial hypercholesterolemia (HeFH) phenotype population and the general population.

	General Population	HeFH Phenotype	*p*
Blood glucose, mg/dL mean (SD)	99 (30)	113 (36)	0.49 (ns)
Systolic blood pressure, mmHg mean (SD)	123 (17)	136 (18)	<0.001
Diastolic blood pressure, mmHg mean (SD)	75 (11)	79 (12)	0.075 (ns)
Baseline total cholesterol, mean (SD)	201 (39)	296 (35)	<0.001
Baseline triglycerides, mean (SD)	136 (65)	128 (43)	0.45 (ns)
Baseline HDL cholesterol, mean (SD)	52 (13)	51 (15)	0.71 (ns)
Baseline LDL cholesterol, mean (SD)	126 (29)	224 (42)	<0.001
Receiving lipid-lowering treatment, *n* (%)	1514 (19.8)	33 (80.5)	<0.001
Treated total cholesterol, mean (SD)	185 (36)	202 (34	0.007
Treated triglycerides, mean (SD)	136 (64)	105 (35)	0.011
Treated HDL cholesterol, mean (SD)	52 (13)	60 (14)	0.002
Treated LDL cholesterol, mean (SD)	108 (33)	130 (53)	0.048

HDL, high-density lipoprotein; LDL, low-density lipoprotein; SD, standard deviation.

## Data Availability

The original contributions presented in this study are included in the article. Further inquiries can be directed to the corresponding author.
